# Subcapsular Renal Hematoma in Simultaneous Pancreas Kidney Transplantation

**DOI:** 10.1155/2020/6152035

**Published:** 2020-05-11

**Authors:** Daniele Cappellani, Chiara Terrenzio, Elena Gianetti, Walter Baronti, Valerio Borrelli, Lorella Marselli, Fabio Vistoli, Alessandro Campatelli, Ugo Boggi, Piero Marchetti

**Affiliations:** ^1^Department of Clinical and Experimental Medicine, Division of Metabolism and Cell Transplantation, University of Pisa, Pisa, Italy; ^2^Division of Diagnostic and Interventional Ultrasound in Transplants, University Hospital of Pisa, Pisa, Italy; ^3^Department of Translational Research and New Technologies in Medicine and Surgery, Division of General and Transplant Surgery, University of Pisa, Pisa, Italy

## Abstract

Subcapsular renal hematoma (SRH) is a challenging condition, which may jeopardize kidney function or constitute a life-threatening event. This is particularly true in single-kidney patients, such as kidney-transplant recipients. SRH may exert an excessive pressure on the surrounding parenchyma, thus resulting in hypoperfusion and ischemia, with high risk of acute kidney failure and graft loss. Moreover, SRH may precede an overt renal rupture with subsequent hemorrhage and hemodynamic instability. The indication to an interventional management for this condition is still a matter of debate, with some authors advocating the high possibilities of spontaneous resolution and others advocating the high-risk of graft loss and even internal bleeding in case of overt renal rupture. Herein, we report the case of a 51-year-old simultaneous pancreas-kidney transplantation recipient who presented a SRH following a mild trauma. The therapeutic choices were carefully balanced on the specific case, and the conservative management proved successful.

## 1. Introduction

Subcapsular renal hematoma (SRH) is a rare although challenging condition that may constitute a life-threatening event. It is defined as a localized collection of blood underneath the renal capsule. SHR may exert excessive pressure on the surrounding parenchyma, causing renal hypoperfusion and refractory hypertension (probably via the inappropriate activation of the renin-angiotensin-aldosterone axis) or sometimes ischemia [1]. Moreover, SRH may precede overt renal rupture with subsequent internal bleeding. SRH is particularly troublesome in single-kidney patients, since it may jeopardize renal function, leading to acute kidney insufficiency. Out of the SRH cases reported in the scientific literature, forty-four reports refer to kidney grafts (Table 1). It is still a matter of debate whether patients diagnosed with SRH should undergo interventional treatments (such as percutaneous drainage, surgical decortication, and nephrectomy) [2–4] or a cautious wait-and-see approach, due to the possible spontaneous resolution of this condition [5, 6]. Therapy should rely on a multidisciplinary approach and should be tailored on the single patient. Herein we report a case of trauma-induced SRH in a simultaneous pancreas-kidney transplantation (SPKT) recipient. Informed consent was obtained from the patient.

## 2. Case Presentation

The patient was a 51-year-old man who received diagnosis of type 1 diabetes mellitus at the age of 13, and underwent successful SPKT for the presence of brittle type 1 diabetes mellitus with severe hypoglycemic episodes and stage-4 chronic kidney disease at our University Hospital when he was 36 years old. The pancreatic-duodenal graft was placed in right iliac fossa, the exocrine drainage was made through a direct anastomosis between donor duodenum and recipient small bowel, and the endocrine drainage was made through a venous anastomosis in the right iliac vein. The renal graft was placed in the left iliac fossa. Maintenance immunosuppressive therapy included tacrolimus, mycophenolic acid, and prednisone. In the postoperative period, both the pancreatic and renal graft maintained normal function, and insulin therapy was dismissed. He underwent regular follow-up as an outpatient at our Department, where there is an active kidney and pancreas transplantation follow-up program [7, 8], for the subsequent fourteen years. During that period HbA1c concentrations remained within the normal reference range and no hypoglycemic episode was reported. Estimated glomerular filtration rate (eGFR, estimated with the Chronic Kidney Disease Epidemiology Collaboration CKD-EPI formula [9]) was stable between 60 and 80 mL/min/1.73 m^2^.

He went to the Emergency Department of a peripheral Hospital for persistent pain in the left iliac fossa. The pain presented following a traumatic episode during a sexual intercourse and underwent a progressive increase in intensity. The patient was referred to our University Hospital for the ultrasound evidence of a subcapsular hematoma on the convex surface of the renal graft.

**Figure 1:**
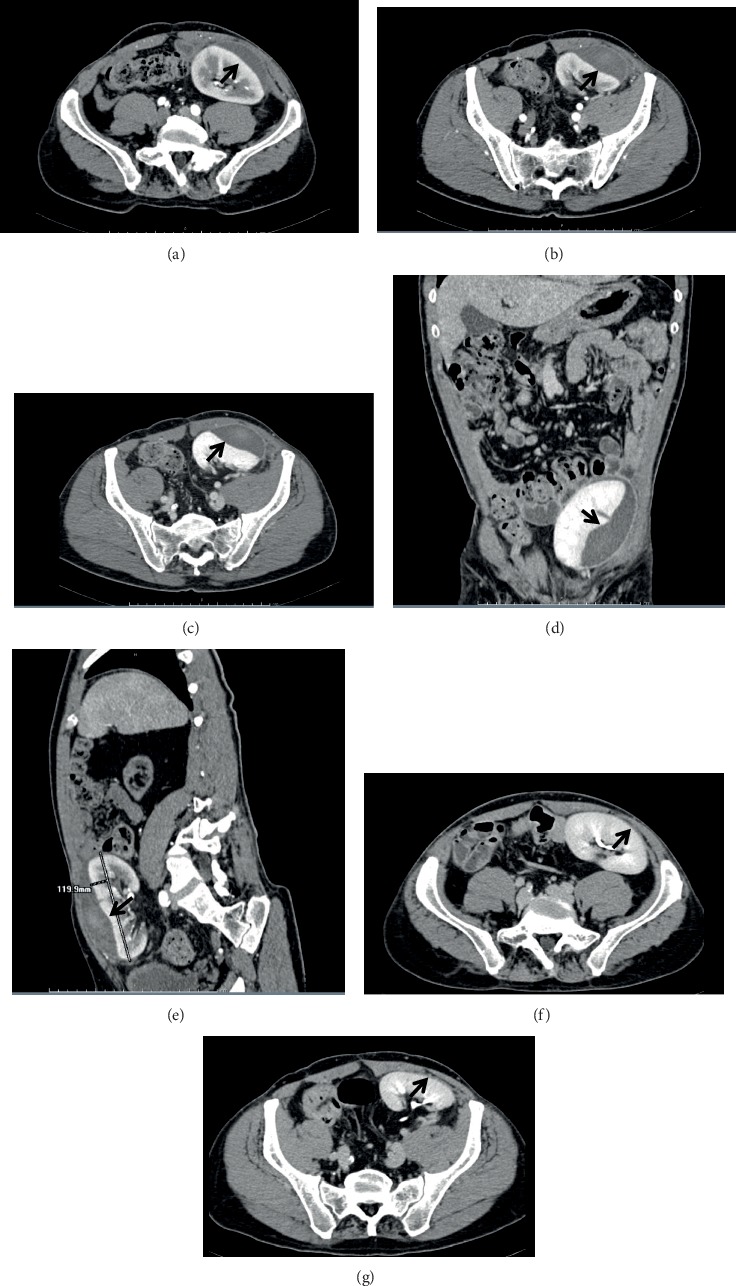
Contrast-enhanced computerized tomography (CT) scans of the transplanted kidney. (a–e) CT scan at admission showing the subcapsular hematoma (arrows). (a, b) Arterial phase. (c) Venous phase. (d) Coronal reconstruction. (e) Sagittal reconstruction. (f, g) CT scan at 4-month follow-up control showing the resolution of the subcapsular hematoma (late arterial phase). In (f, g), the arrows indicate the site of the previous hematoma.

Physical examination at admission was unremarkable except for mild pain and tenderness of the left iliac fossa. Blood pressure was 125/85 mmHg. Biochemical findings were normal or stable compared to previous evaluations: creatinine 1.34 mg/dL, eGFR 60.9 mL/min/1.73 m^2^, glucose 98 mg/dL, HbA1c 38 mmol/mol, hemoglobin 14.7 g/dL, white blood cells 11.72 × 10^3^/*μ*L. Contrast-enhanced computerized tomography showed a subcapsular hematoma of 6.5 × 3 × 12 cm with regular urinary outflow, without signs of contrast leakage outside the vascular bed (Figures 1(a)–1(e)).

Taken into account the overall stable clinical conditions of the patient and the results of the tests performed, we alerted the kidney transplant surgeons and decided to adopt a wait-and-see approach. The patient was therefore maintained under strict observation.

**Figure 2:**
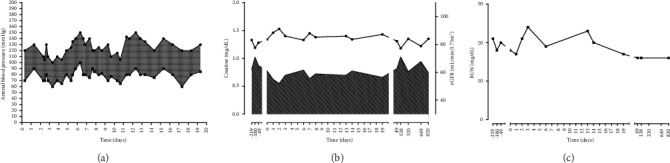
Clinical and biochemical parameters during the hospital stay. (a) Systolic and diastolic arterial blood pressure. (b) Creatinine concentration and estimated glomerular filtration rate. (c) Blood urea nitrogen concentration.

**Figure 3:**
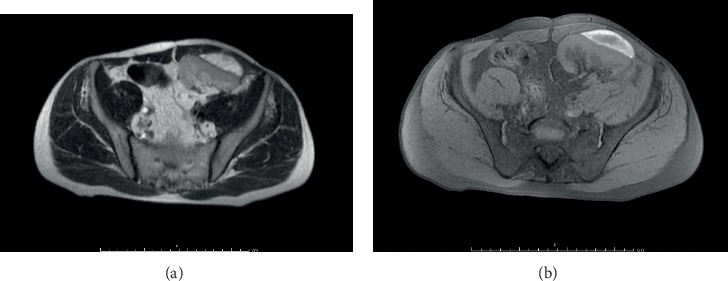
Contrast-enhanced magnetic resonance imaging at day 9 showing the dimensional reduction of the subcapsular hematoma in the transplanted kidney and the absence of fluids in the perirenal spaces. (a) Axial T2-weighted. (b) Axial T1-weighted.

Renal function, urinary output, and blood pressure remained stable and within the normal range during the whole hospital stay (Figure 2), and the SRH underwent progressive reduction. At day 9, a contrast-enhanced magnetic resonance reported the SRH dimensional reduction and the absence of fluids in the perirenal space (Figure 3). The complete resolution of the SRH was demonstrated by abdominal ultrasound after 19 days and confirmed at a CT-scan performed 4 months later (Figures 1(f) and 1(g)). The patient underwent subsequent regular follow-up in the outpatient setting: pancreatic and renal function remained stable and renal imaging showed the normality of the renal graft 3 years after the hospitalization.

## 3. Discussion

SRH is more often derived from traumatic accidents. Other common causes of subcapsular hematomas include medical procedures, such as renal biopsy and shock-wave lithotripsy.

SRH is a challenging condition since it may herald different unfavorable events. As a matter of fact, SRH presentation may vary widely, depending on the hematoma dimensions, the compression exerted over the surrounding parenchyma and the blood losses. The hematoma-exerted pressure on the surrounding parenchyma may induce hypoperfusion and sometimes refractory hypertension [1], a phenomenon named Page kidney disease. This condition is named after the first report of refractory hypertension caused by external renal compression made by Irvine Page in 1939 [10]. In this setting, arterial hypertension is thought to be due to the activation of the renin-angiotensin-aldosterone axis as a consequence of the hematoma-induced pressure on the renal parenchyma.

Moreover, SRH may precede overt renal rupture, with internal bleeding and hemodynamic instability [4]. Renal rupture has an incidence of 0.3-9.6% in kidney transplant series [11]. Renal graft rupture is usually an early event set in the postoperative period [12], and its most important cause is acute organ rejection, followed by renal vein thrombosis, acute tubular necrosis, trauma, renal biopsy, ureteral obstruction, and cancer. The clinical presentation of kidney rupture is often dramatic, due to acute blood loss and severe graft dysfunction, which may, respectively, lead to hemodynamic instability and multiple organ failure [13, 14].

Furthermore, SRH may result in deterioration of renal function. This point may be particularly troublesome in patients with a baseline reduced renal function, such as patients with chronic kidney disease or single-kidney patients. In such circumstances, SRH may jeopardize renal function, sometimes resulting in acute kidney failure.

It is still debated whether SRH should undergo interventional (such as percutaneous drainage, surgical decortication, and nephrectomy) [2] or wait-and-see approach, based on the possible spontaneous resolution of this condition [5]. In the event of overt kidney rupture, the conservative management has a low success rate [15], whereas the salvage rate of surgery is estimated to be 80%, with long-term outcomes similar to the general population [16]. The conservative management of the Page kidney phenomenon includes drugs acting on the renin-angiotensin-aldosterone system (i.e., ACE inhibitors and angiotensin receptor blockers). If medical therapies do not control arterial hypertension or if renal function significantly decreases, more invasive options (such as percutaneous drainage and surgical decompression) should be taken into account [2].

To the best of our knowledge, this is the first report of SRH in a SPKT recipient. In this case, a conservative approach proved successful, leading to the resolution of the hematoma without invasive procedures. This strategy required a careful noninvasive monitoring in the presence of surgical expertise readily available in case of need. Pros and cons of conservative and interventional approaches should be carefully weighted, and the decision-process should be tailored on the specific case. In this specific situation, the additional presence of a pancreatic graft did not influence significantly our therapeutic choices, being the immunosuppressive regimen carefully followed and the glycemic control stable. However, the presence of two different grafts, both of which should possibly be preserved, should be considered when taking decisions about the management of SHR.

## Figures and Tables

**Table 1:** Reports of subcapsular renal hematoma (SRH) published so far.

Reference	Number of patients	Cause of the SRH	Management	Outcome
Figueroa TE et al., J Urol. 1988 Aug;140 (2):355-6	1	Biopsy	Surgical (decompression)	Complete resolution
Kliewer MA et al., Radiographics. 1991 Mar; 11 (2):336-7	1	Biopsy	Surgical (nephrectomy)	Graft loss
Dempsey J et al., South Med J. 1993 May; 86 (5):574-7	1	Biopsy	Surgical (decompression)	Complete resolution
Nguyen BD et al., Clin Nucl Med. 1994 Apr; 19 (4):361-3	1	Following transplantation	Surgical (decompression)	Complete resolution
Goyal M et al., Clin Nucl Med. 1996 Apr; 21 (4):345-6	1	Trauma	NA	NA
Machida J et al., Int J Urol. 1996 May; 3 (3):228-30	1	Biopsy	Conservative	Partial resolution
Tanabe K et al., J Urol. 1998 Sep; 160 (3 Pt 2):1212-5	1	Following transplantation	Surgical (decompression)	Complete resolution
Rea R et al., Nephrol Dial Transplant. 2000 Jul; 15 (7):1104-5	1	Biopsy	Surgical (decompression)	Complete resolution
Gibney EM et al., Transplantation. 2005 Jul 27; 80 (2):285-6	1	During transplantation	Surgical (decompression)	Complete resolution
Patel TV et al., Kidney Int. 2007 Dec; 72 (12):1562	1	Biopsy	Failed conservative attempt and subsequent surgical management (decompression)	Complete resolution
Chung J et al., Am J Transplant. 2008 Jun; 8 (6):1323-8	4	Biopsy	4/4 Surgical (decompression)	3/4 Complete resolution 1/4 Graft loss
Caldés S et al., Transplantation. 2009 Jan 27; 87 (2):303-4	1	Nephrostomy	Failed percutaneous drainage attempt and subsequent surgical management (decompression)	Complete resolution
Kamar N et al., Transplantation. 2009 Feb 15; 87 (3):453-4	2	Biopsy	2/2 Conservative	2/2 Complete resolution
Basaran C et al., Clin Radiol. 2009 May; 64 (5):523-8	1	Acute rejection	Surgical (nephrectomy)	Graft loss
Heffernan E et al., J Clin Ultrasound. 2009 May; 37 (4):226-9	1	Biopsy	Surgical (decompression)	Complete resolution
Salgado, OJ et al., J Clin Ultrasound. 2010 Mar-Apr; 38 (3):164-7	1	During transplantation	Conservative	Complete resolution
Posadas MA et al., Scientific World Journal. 2010 Aug 3; 10 : 1539-42	1	Biopsy	Surgical (decompression)	Complete resolution
Friedersdorff F et al., Transplant Proc. 2010 Nov; 42 (9):3868-70	1	Lithotripsy	Conservative	Complete resolution
Butt FK et al., Transplant Proc. 2010 Dec; 42 (10):4291-4	1	Spontaneous	Surgical (decompression)	Complete resolution
Okechukwu O et al., Saudi J Kidney Dis Transpl. 2011 Jul; 22 (4):796-8	1	Following transplantation	Surgical (decompression)	Complete resolution
Thiyagarajan UM et al., Int J Surg Case Rep. 2011; 2 (7):188-90	1	Biopsy	Surgical (decompression)	Complete resolution
Maurya KK et al., Saudi J Kidney Dis Transpl. 2011 Sep; 22 (5):1012-3	1	Biopsy	Surgical (decompression)	Complete resolution
Gandhi V et al., BMJ Case Rep. 2012 Dec 6;2012. pii: bcr2012007653	1	Spontaneous	Surgical (decompression)	Complete resolution
Hamidian JA et al., Iran J Kidney Dis. 2013 Sep; 7 (5):352-5	1	Renal artery stenting	Percutaneous drainage	Complete resolution
Adjei-Gyamfi Y et al. Pediatr Transplant. 2014 Dec; 18 (8):E262-5	2	Biopsy	2/2 Surgical (decompression)	2/2 Complete resolution
Kumar A et al., Clin Nephrol Case Stud. 2015 May 22; 3 : 5-7	1	Trauma	Failed conservative attempt and subsequent surgical management (decompression)	Complete resolution
Kapoor R et al., Case Rep Med. 2016; 2016 : 3898307	1	Acute renal failure	Failed percutaneous drainage attempt and subsequent surgical management (decompression)	Complete resolution
